# Supplementary material for: The roles of nuclear orphan receptor NR2F6 in anti-viral innate immunity
Source: PLoS Pathog. 2024 Jun 3;20(6):e1012271. doi: 10.1371/journal.ppat.1012271 (PMC11175508; doi:10.1371/journal.ppat.1012271)
Supplement: S1 Fig — Effects of deficient Mef2d (A) and Maff (B) on HSV-1 transcription in MEF cells. The MEF cells were treated with siRNA for 48 h before infection with HSV-1. The MEF cells were infected with or without HSV-1 (MOI = 1) for 8h before qPCR analysis. (C) Effects of deficient NR2F6 on HSV-1 in THP-1. The THP-1 cells were infected with different virus titers for 24h (MOI = 0.25, 0.5, 1). (D) Effects of deficient NR2F6 on HSV-1 in THP-1. The THP-1 cells were infected with HSV-1(MOI = 1) for different times (8h, 24h). Graphs show mean ± SEM, n = 3. **P < 0.01, *P < 0.05. (PDF) [file ppat.1012271.s001.pdf]

Sup. Fig. S1

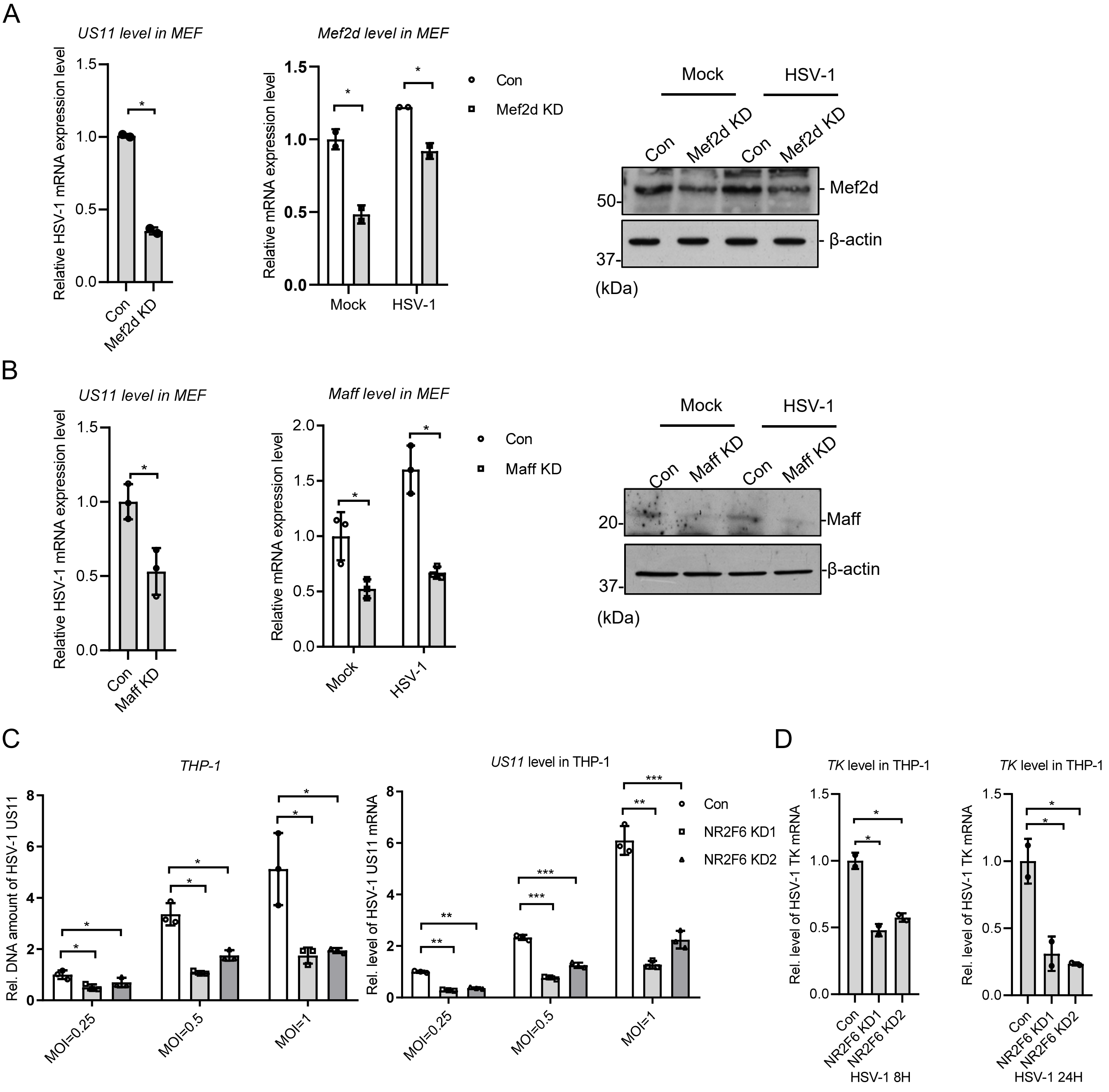

Sup. Fig. S1 Other anti-virus transcription factor candidates affected HSV-1 transcription. **(A&B)** Effects of deficient Mef2d (A) and Maff (B) on HSV-1 transcription in MEF cells. The MEF cells were treated with siRNA for 48 h before infection with HSV-1. The MEF cells were infected with or without HSV-1 (MOI = 1) for 8h before qPCR analysis. **(C)** Effects of deficient NR2F6 on HSV-1 in THP-1. The THP-1 cells were infected with different virus titers for 24h (MOI=0.25, 0.5, 1). **(D)** Effects of deficient NR2F6 on HSV-1 in THP-1. The THP-1 cells were infected with HSV-1(MOI=1) for different times (8h, 24h). Graphs show mean ± SEM, n = 3. **P < 0.01, *P < 0.05.
