# Supplementary material for: The roles of nuclear orphan receptor NR2F6 in anti-viral innate immunity
Source: PLoS Pathog. 2024 Jun 3;20(6):e1012271. doi: 10.1371/journal.ppat.1012271 (PMC11175508; doi:10.1371/journal.ppat.1012271)
Supplement: S2 Fig — Effects of deficient NR2F6 on HSV-1 and SeV transcription in U2OS(A) and HFF(B). The cells were transfected with siRNA for 48h before infection with HSV-1 or SeV. The cells were left uninfected or infected with HSV-1 (MOI = 1) for 8h or SeV for 12h before qPCR analysis. Effects of overexpressed NR2F6 on SeV transcription in MEF(C) and HEK293T(D). The cells were transfected with plasmids for 48h before infection with SeV. The cells were left uninfected or infected with SeV for 12h before analysis. (E) Effects of deficient Nr2f6 on MCMV transcription and replication in MEF. The MEF cells were left uninfected or infected with MCMV (MOI = 1) for 72h before qPCR analysis. Graphs show mean ± SEM, n = 3. **P < 0.01, *P < 0.05. (PDF) [file ppat.1012271.s002.pdf]

Sup. Fig. S2

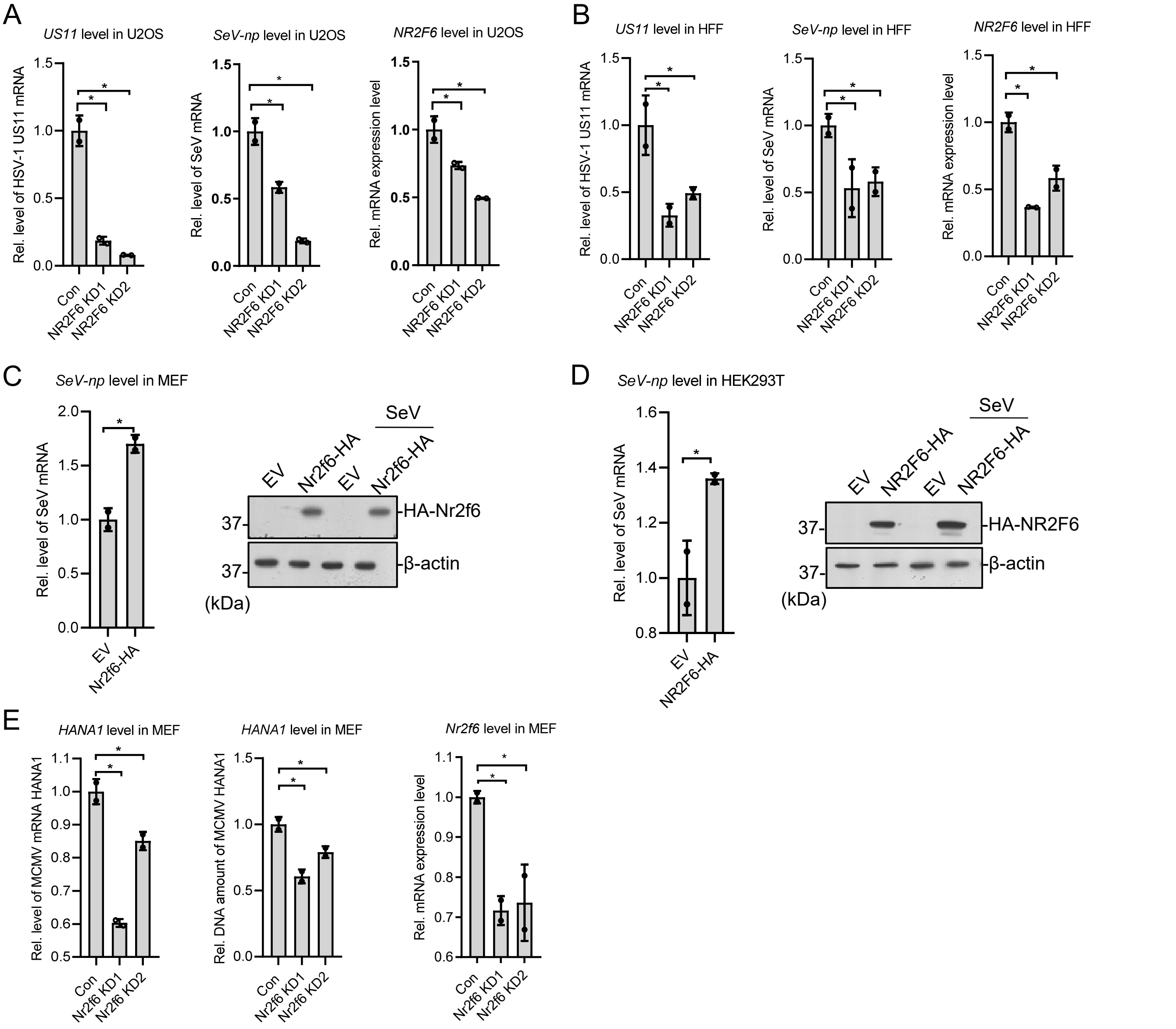

Sup. Fig. S2 NR2F6 can affect the transcription and replication of multiple viruses in a variety of cells. Effects of deficient NR2F6 on HSV-1 and SeV transcription in U2OS**(A)** and HFF**(B)**. The cells were transfected with siRNA for 48h before infection with HSV-1 or SeV. The cells were left uninfected or infected with HSV-1 (MOI = 1) for 8h or SeV for 12h before qPCR analysis. Effects of overexpressed NR2F6 on SeV transcription in MEF**(C)** and HEK293T**(D).** The cells were transfected with plasmids for 48h before infection with SeV. The cells were left uninfected or infected with SeV for 12h before analysis. **(E)** Effects of deficient Nr2f6 on MCMV transcription and replication in MEF. The MEF cells were left uninfected or infected with MCMV (MOI = 1) for 72h before qPCR analysis. Graphs show mean ± SEM, n = 3. **P < 0.01, *P < 0.05.
