# Supplementary material for: The roles of nuclear orphan receptor NR2F6 in anti-viral innate immunity
Source: PLoS Pathog. 2024 Jun 3;20(6):e1012271. doi: 10.1371/journal.ppat.1012271 (PMC11175508; doi:10.1371/journal.ppat.1012271)
Supplement: S3 Fig — (A)The Nr2f6 gene in mice were mutated using CRISPR system. (B) The genome identification results in different genotypes. (C) Transcription level of Nr2f6 in different mouse genotypes. The weight of spleen(D), lung (E) and liver (F) of wide type and Nr2f6+/- mice was measured. (PDF) [file ppat.1012271.s003.pdf]

Sup. Fig. S3

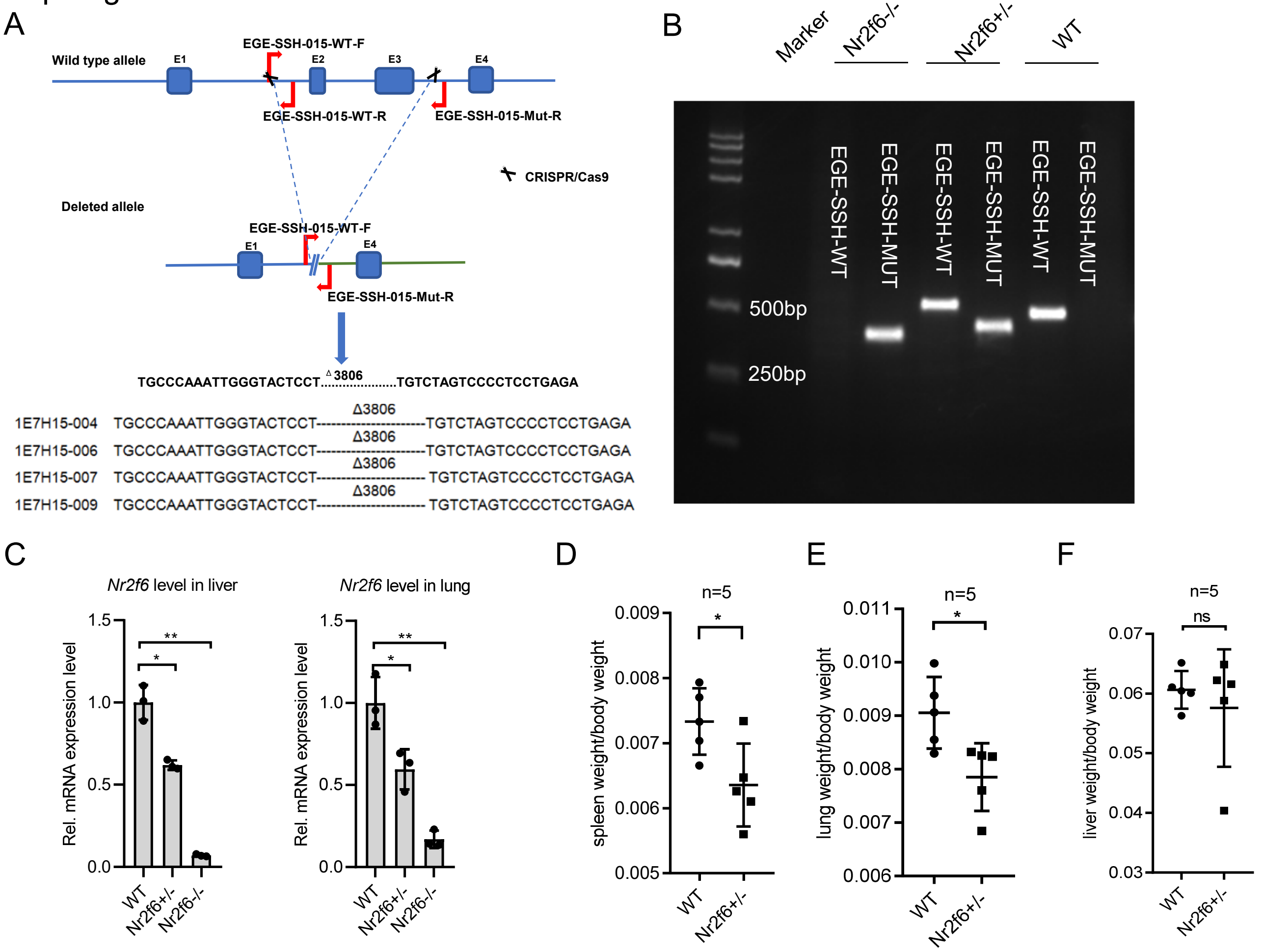

Sup. Fig. S3 The impact of Nr2f6 deficiency on mouse development. (A) The Nr2f6 gene in mice were mutated using CRISPR system. **(B)** The genome identification results in different genotypes. **(C)** Transcription level of Nr2f6 in different mouse genotypes. The weight of spleen**(D),** lung **(E)** and liver **(F)** of wide type and Nr2f6$^{+/-}$ mice was measured.
