# Supplementary material for: The roles of nuclear orphan receptor NR2F6 in anti-viral innate immunity
Source: PLoS Pathog. 2024 Jun 3;20(6):e1012271. doi: 10.1371/journal.ppat.1012271 (PMC11175508; doi:10.1371/journal.ppat.1012271)
Supplement: S4 Fig — (A) Effects of NR2F6 deficiency on transcription of downstream genes(FOS, IL8, ISG56) induced by HSV-1 in THP1 cells. The THP1 cells were infected with HSV-1 (MOI = 1) for 24 h before qPCR analysis. (B) Effects of overexpressed Nr2f6 on TBK1-IFNB1 and JNK passway in MEF cells. The cells were transfected with plasmids for 48 h before infection with HSV-1. The cells were left uninfected or infected with HSV-1 (MOI = 1) for 8 h before immunoblotting analysis. (C) Effects of overexpressed NR2F6 on transcription of downstream genes(IFNB1, ISG54, ISG56) induced by HSV-1 in THP1 cells. The THP1 cells were infected with HSV-1 (MOI = 1) for 24 h before qPCR analysis. Graphs show mean ± SEM, n = 3. **P < 0.01, *P < 0.05. (PDF) [file ppat.1012271.s004.pdf]

Sup. Fig. S4

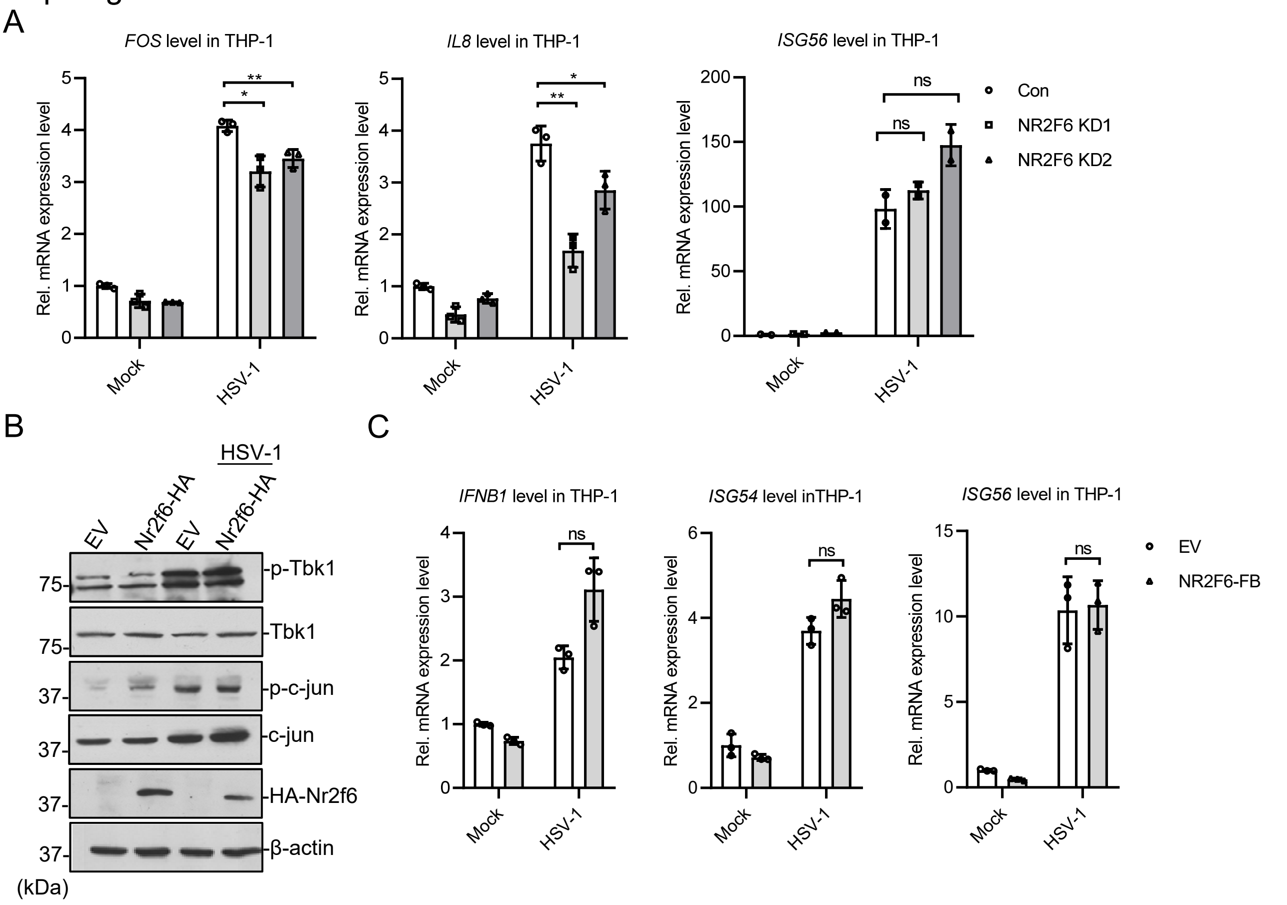

Sup. Fig. S4 Effects of NR2F6 on gene transcription of anti-virus innate immunity. (A) Effects of NR2F6 deficiency on transcription of downstream genes （*FOS, IL8, ISG56*) induced by HSV-1 in THP1 cells. The THP1 cells were infected with HSV-1 (MOI = 1) for 24 h before qPCR analysis. **(B)** Effects of overexpressed Nr2f6 on TBK1-IFNB1 and JNK passway in MEF cells. The cells were transfected with plasmids for 48 h before infection with HSV-1. The cells were left uninfected or infected with HSV-1 (MOI=1) for 8 h before immunoblotting analysis. **(C)** Effects of overexpressed NR2F6 on transcription of downstream genes （*IFNB1, ISG54, ISG56*) induced by HSV-1 in THP1 cells. The THP1 cells were infected with HSV-1 (MOI = 1) for 24 h before qPCR analysis. Graphs show mean ± SEM, n = 3. **P < 0.01, *P < 0.05.
