# Supplementary material for: The roles of nuclear orphan receptor NR2F6 in anti-viral innate immunity
Source: PLoS Pathog. 2024 Jun 3;20(6):e1012271. doi: 10.1371/journal.ppat.1012271 (PMC11175508; doi:10.1371/journal.ppat.1012271)
Supplement: S5 Fig — (A) Effects of deficient c-Jun on anti-virus innate immunity genes transcription with HSV-1 infection. The THP1 cells were infected with HSV-1 (MOI = 1) for 24 h before qPCR analysis. (B) Effects of deficient c-Jun on SeV replication and anti-virus innate immunity genes (IFNB1, ISG54, ISG56) transcription with SeV infection. The THP1 cells were infected with SeV for 12 h before qPCR analysis. Graphs show mean ± SEM, n = 3. **P < 0.01, *P < 0.05. (PDF) [file ppat.1012271.s005.pdf]

Sup. Fig. S5

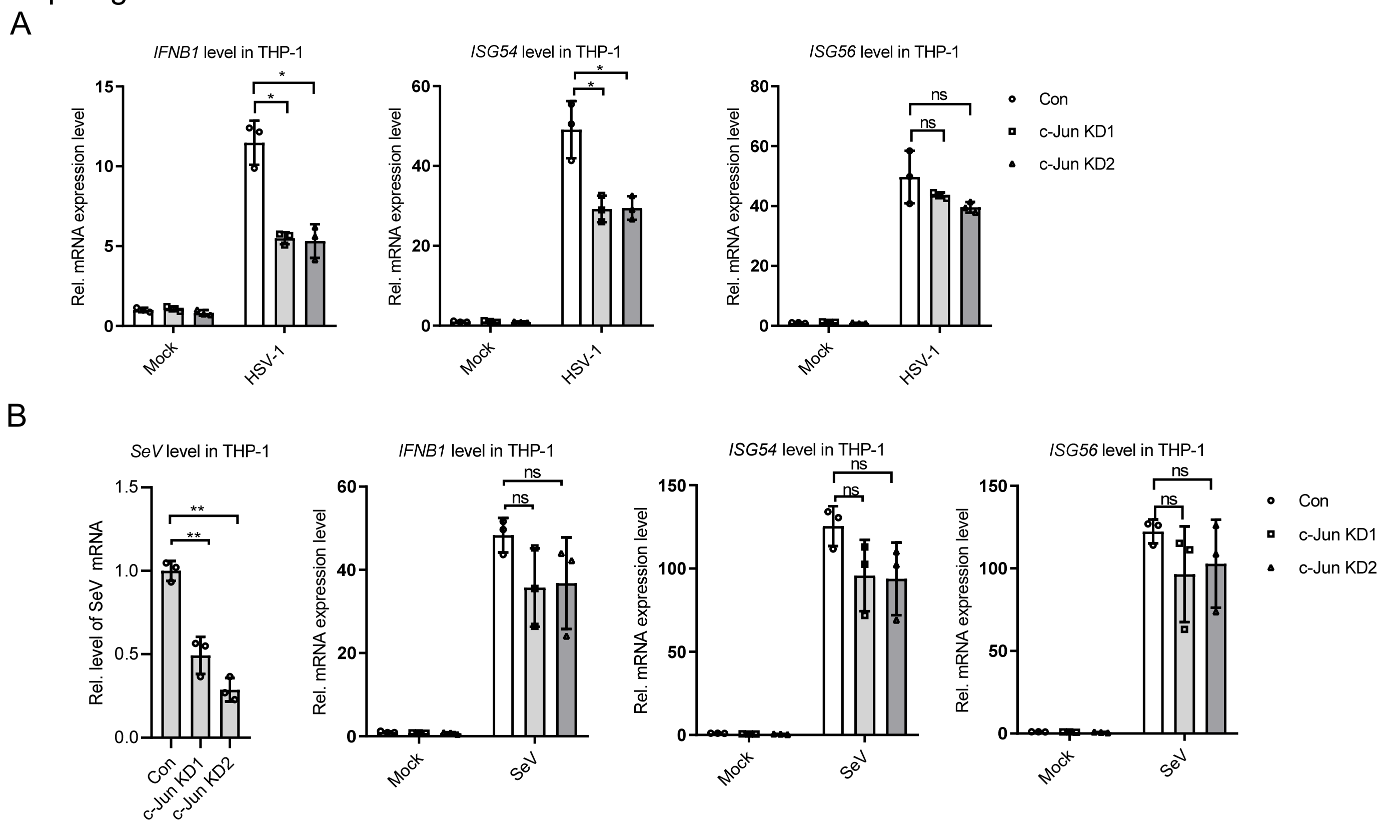

Sup. Fig. S5 AP-1 affected gene transcription of anti-virus innate immunity. (A) Effects of deficient *c-Jun* on anti-virus innate immunity genes transcription with HSV-1 infection. The THP1 cells were infected with HSV-1 (MOI = 1) for 24 h before qPCR analysis. **(B)** Effects of deficient *c-Jun* on SeV replication and anti-virus innate immunity genes (*IFNB1, ISG54, ISG56*) transcription with SeV infection. The THP1 cells were infected with SeV for 12 h before qPCR analysis. Graphs show mean ± SEM, n = 3. **P < 0.01, *P < 0.05.
