# Supplementary material for: The roles of nuclear orphan receptor NR2F6 in anti-viral innate immunity
Source: PLoS Pathog. 2024 Jun 3;20(6):e1012271. doi: 10.1371/journal.ppat.1012271 (PMC11175508; doi:10.1371/journal.ppat.1012271)
Supplement: S6 Fig — (A) NR2F6 affected the transcription of UBC in THP-1 cells. The THP1 cells were infected with HSV-1 (MOI = 1) for 24 h before qPCR analysis. (B) The UCSC genome browser view showed that NR2F6 was enriched on the UBC promoter region. (C) The enrichment of NR2F6 on UBC promoter region was detected in NR2F6-FB overexpressed THP-1 cells by ChIP-qPCR. The THP-1 cells were infected with HSV-1 (MOI = 1) for 24 h before analysis. (D) NR2F6 affected the transcription of TRIB1 in THP-1 cells. The THP1 cells were infected with HSV-1 (MOI = 1) for 24 h before qPCR analysis. (E) The UCSC genome browser view showed that NR2F6 was enriched on the TRIB1 promoter region. (F) The enrichment of NR2F6 on TRIB1 promoter region was detected in NR2F6-FB overexpressed THP-1 cells by ChIP-qPCR. The THP-1 cells were infected with HSV-1 (MOI = 1) for 24 h before analysis. Graphs show mean ± SEM, n = 3. **P < 0.01, *P < 0.05. (PDF) [file ppat.1012271.s006.pdf]

Sup. Fig. S6

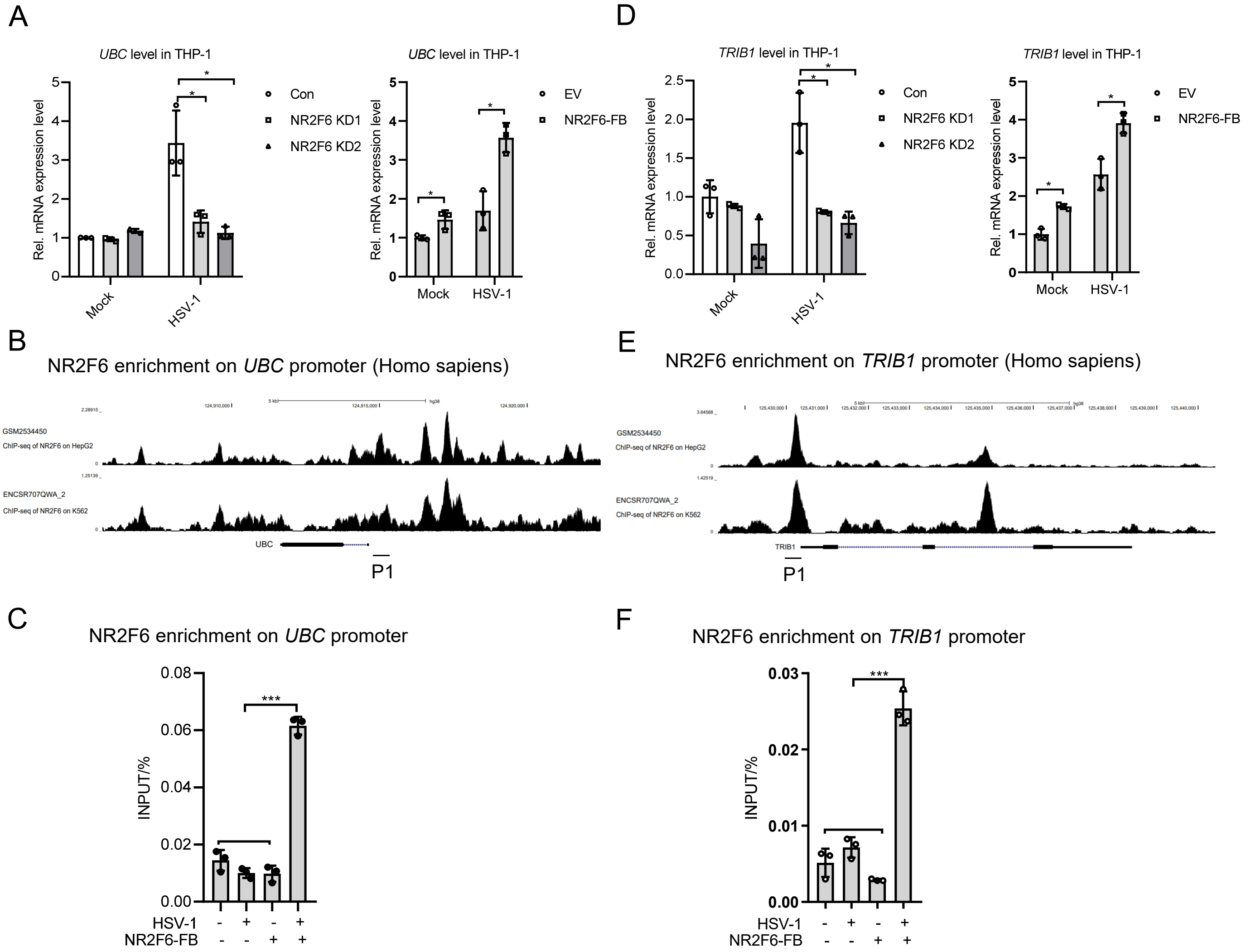

Sup. Fig. S6 NR2F6 directly regulates the transcription of *UBC* and *TRIB1*. (A) NR2F6 affected the transcription of *UBC* in THP-1 cells. The THP1 cells were infected with HSV-1 (MOI = 1) for 24 h before qPCR analysis. **(B)** The UCSC genome browser view showed that NR2F6 was enriched on the *UBC* promoter region. **(C)** The enrichment of NR2F6 on *UBC* promoter region was detected in NR2F6-FB overexpressed THP-1 cells by ChIP-qPCR. The THP-1 cells were infected with HSV-1 (MOI = 1) for 24 h before analysis. **(D)** NR2F6 affected the transcription of *TRIB1* in THP-1 cells. The THP1 cells were infected with HSV-1 (MOI = 1) for 24 h before qPCR analysis. **(E)** The UCSC genome browser view showed that NR2F6 was enriched on the *TRIB1* promoter region. **(F)** The enrichment of NR2F6 on *TRIB1* promoter region was detected in NR2F6-FB overexpressed THP-1 cells by ChIP-qPCR. The THP-1 cells were infected with HSV-1 (MOI = 1) for 24 h before analysis. Graphs show mean ± SEM, n = 3. **P < 0.01, *P < 0.05.
