# Supplementary material for: The roles of nuclear orphan receptor NR2F6 in anti-viral innate immunity
Source: PLoS Pathog. 2024 Jun 3;20(6):e1012271. doi: 10.1371/journal.ppat.1012271 (PMC11175508; doi:10.1371/journal.ppat.1012271)
Supplement: Attachment — Submitted filename: Response.docx [file ppat.1012271.s010.docx]

To whom it may concern,

Thank you for giving us a chance to submit our revised manuscript, titled “*The roles of nuclear orphan receptor NR2F6 in anti-viral innate immunity*” (PPATHOGENS-D-24-00152). We have made all the corrections as suggested. Below is our point-by-point response to each comment, in which the text in blue is the original comments from the reviewers and our response in black.

Reviewer's Responses to Questions

Part I - Summary

Please use this section to discuss strengths/weaknesses of study, novelty/significance, general execution and scholarship.

Reviewer #1: In this study, Yang et al. reports NR2F6 as a new DNA viral infection transcriptional regulator in promoting DNA viral replication. Using RNA-Seq and H3K27ac ChIP-Seq in THP1 cells upon HSV-1 infection, the authors found enriched TF motifs upon viral infection. By validating hits, the authors found NR2F6 facilitated DNA viral replication. By generating Nr2f6 hetero-mice, the authors confirmed a positive regulator role of Nr2f6 in facilitating viral replication in vivo. Further mechanistic studies reveal involvement of JNK/c-Jun with Nr2f6 in regulating MAP3K5 transcription, while a negative suppression loop by IRF3/STAT3 has also been observed. Overall, the authors provide ample amount of data to indicate the presence of a new NR2F6 transcriptional program in facilitating DNA viral infection. However, there are severe technical concerns and some conceptual concerns that preclude the study to be considered for publication.

Response: We appreciate the review’s comments to our work, and we have made corrections to address the concerns, which are listed below.

Technical concerns: Inconsistent data have been presented across the data panels that reduce the confidence of the reviewer on this study. eg. NR2F6 mRNA levels are not consistent for with/without HSV-1 infection in Fig. 1F (decreased upon HSV-1) vs Fig. 7F (no significance); the NR2F6 western blots in Fig. 6B (doublets) vs. Fig. 6D and many other panels (single band).

Response: As we have shown in Fig. 6B, the mRNA level of NR2F6 decreases soon after HSV-1 treatment and recovers at 24 hr after infection. So, the time point to harvest cells for analysis determines whether the reduction of NR2F6 can be observed. In Fig. 7F, the cells were harvested 24 hr after infection and that’s why we did not observed NR2F6 decrease after HSV-1 treatment.

When we detect the protein level of endogenous NR2F6 with the antibody we have, we could often see two bands with long exposure, and only one band with short exposure. In Fig. 5C, we have shown that the major NR2F6 forms in cytosol and nuclei are different in molecular weight. It is highly possible that two different forms of NR2F6 exist in the cells, but currently we do not know whether they are different isoforms or with different modifications. Future studies are required to further elucidate the problem.

Conceptual concerns: If NR2F6 is facilitating DNA viral replication and infection, its suppression by STAT3 should occur in later infection stage- why at 24 hr infection increased NR2F6 genome localization is observed (by H3K27Ac) but the overall NR2F6 expression is reduced? And time-dependent NR2F6 has not been established.

Response: We feel it is maybe some misunderstanding here. We predicted transcription factors using both up-regulated and down-regulated enhancers. NR2F6 was predicted from the motifs of down-regulated enhancers, and it was consistent with the reduction of NR2F6 level. The time-dependent level of NR2F6 has been shown in Fig. 6B.

Reviewer #2: I think this work is generally robust, interesting, and important. My biggest qualms about this manuscript have to do with the presentation of the story and data, as well as physiological relevance of some of the cell lines used. Especially knowing how far animal models and neuronal cell culture models have come for HSV work, I found the models to be a little underwhelming.

Strengths – multiple orthogonal approaches identified pro-viral transcription factors not previously appreciated. Mapped mechanism to signaling pathway, and changes in genome occupancy of transcription factors that underlie the phenotypes presented.

Weaknesses – despite the amount of compelling data, it was difficult to understand due to the way the data were presented (see minor issues below).

Response: We appreciate the reviewer for his/her positive comment. We have also try our best to correct our manuscript to address the concerns.

Reviewer #3: In this manuscript, Yang et. al unravel a complex web of regulation centered around a transcription factor, NR2F6, that they have uncovered as part of the response to viral infection using HSV-1 as a model. First, they perform RNA-seq to identify upregulated differentially expressed genes during HSV-1 infection, then performed H3K27ac-ChIP-seq to identify active enhancers within this group. From this dataset, potential transcription factors were identified and several (Nr2f6, Mef2d and Maff) were characterized. This paper focuses on NR2F6, an orphan nuclear receptor. The authors show that knockdown of NR2F6 reduces HSV-1 replication and that overexpression enhances replication. The authors then generate Nr2f6+/- mice and show that partial loss of NR2F6 reduces viral replication in isolated BMDMs and mice. RNA-seq of NR2F6-null cells revealed that differentially expressed genes were enriched in the JNK pathway.

The authors going on to show that NR2F6 promotes HSV-1 replication not through the cGAS/STING pathway, but instead through the JNK pathway. NR2F6 works with c-Jun to bind to the MAP3K5 promoter, activating the JNK pathway. NR2F6 is in turn downregulated by both STAT3 (cGAS/STING) and the AP-1/c-Jun pathway.

The work is novel, unveiling a new piece of the innate immune system that responds to viral infection. Although HSV-1 was used in this paper, similar trends hold for MCMV and Sendai virus, suggesting that this pathway may be broadly involved in viral infection. The work is thorough and shows similar trends in vitro in many different cell types and in vivo.

Response: We appreciate the reviewer for his/her positive comment.

Part II – Major Issues: Key Experiments Required for Acceptance

Please use this section to detail the key new experiments or modifications of existing experiments that should be absolutely required to validate study conclusions.

Generally, there should be no more than 3 such required experiments or major modifications for a "Major Revision" recommendation. If more than 3 experiments are necessary to validate the study conclusions, then you are encouraged to recommend "Reject".

Reviewer #1: Specific comments:

1. The knockdown efficiency of Med2d in Fig. S1A is quite poor thus conclusions from Med2d are not conclusive.

Response: we have repeated the experiments and replaced them with new figures (Response Fig. 1).

2. Fig 1F: since Nr2f6 is the major target for this study, protein level changes would also need to be monitored in this assay in addition to mRNA changes. In addition, the statistical analysis in Nr2f6 levels between blank and HSV-1 infection groups in con should be performed- does this suggest HSV-1 infection at 24 hr time point suppresses Nr2f6 transcription? If so, how can Nr2f6 mediated enhancer-mediated transcription be activated and enriched?- also the authors should comment on why in Fig. S1A Med2d mRNA levels increased upon HSV-1 infection, which is opposite to Fig. 1F.

Response: Thank you for the suggestions. We are willing to add the WB result for Nr2f6 KD into the figures. Unfortunately, the antibody we have does not recognize mouse Nr2f6, so we could only check Nr2f6 mRNA in MEF cells. For those experiments done in human cells, we have provided WB for NR2F6 (Fig. 2A, 3D, 3E, 4A, 5A, 5C, 6B, 6C, 7A, 7D). I hope the reviewer could understand our difficulty.

Response Fig. 1 Verification of Mef2D and Maff in regulating HSV-1. **(A&B)** Effects of deficient Mef2d (A) and Maff (B) on HSV-1 transcription in MEF cells. The MEF cells were treated with siRNA for 48h before infection with HSV-1. The MEF cells were infected with or without HSV-1 (MOI = 1) for 8 h before qPCR analysis.

We have added statistical analysis for Fig. 1F as suggest. In Fig. 1F, cells were harvested 8 hr after HSV-1 infection, not 24 hr. The result is consistent with that in Fig.6B, Nr2f6 mRNA was repressed 8 hr after infection. For the NR2F6 target enhancers, we guess the reviewer may mis-understand our pipeline. During bioinformatic analysis, we first identified all the active enhancers in control and HSV-1-treated cells, and compared the two groups to get up-regulated and down-regulated enhancers. Then we predicted transcription factors based on both the up- and down-regulated enhancers. NR2F6 was predicted from the down-regulated enhancers, which are shown in the right part of Fig. 1D. NR2F6 belongs to the same protein family of NR2F2, and the software only present the representative family member here.

We have repeated the experiment for Mef2d, the mRNA and protein levels of Mef2d actually did not show significant increase after HSV-1 treatment. We have replaced the figures in Sup Fig. S1. In Fig. 1F, we did not show any result for Mef2d, and we think the reviewer probably refer to Fig. 1E here. Fig. 1E showed that the mRNA of MEF2D decreased after HSV-1 infection in THP-1, but we did not observe it in MEF. The disagreement is possible due to the different cell lines used in the two studies.

3. Fig. 1G: these blots were not aligned well.

Response: We have rearranged the figures as suggested.

4. One major concern for NRF2D6 knockdown is if this affects cell proliferation- if so proliferation changes would expect to affect viral infection. Thus, at least cell viability or colony formation assays should be performed in mouse and human lines used here.

Response: We have measured the proliferation of control and Nr2f6-FB stable-expressed MEF cells with colony formation assay, or counted the cell numbers of THP-1 cells with NR2F6-FB stable expression or knockdown. No significant difference was observed in these cell lines (Response Fig. 2). So, we think NR2F6 expression or knockdown does not affect cell proliferation in the tested cell lines.

**Response Fig. 2 Effect of NR2F6 on cell proliferation. (A)** FB-tagged Nr2f6 was stably expressed in MEF cells. Cell proliferation was assayed by colony formation assay. **(B)** FB-tagged NR2F6 was stably expressed in THP-1 (left), or NR2F6 was knocked down in THP1 using CRISPR technique. Cell number was counted manully every 24 hr to measure cell proliferation.

5. Fig S3: how about the body weights for normal and hetero- mice?

Response: We did not observe significant difference of body weights between the two groups (Response Fig. 3).

Response Fig. 3 Body weight of wild type and *Nr2f6*^+/-^ mice (n = 5).

6. Fig 2B: NR2F6 signals are too weak to find endogenous signals.

Response: We have replaced it with a new figure (Response Fig. 4).

Response Fig. 4 The lysates of the control and *NR2F6* stable expressed THP-1 cells were blotted with anti-NR2F6 antibody.

7. Fig 2C-2F: one of the major HSV-1 infection organ is brain, thus brain viral load should be monitored. What is the rationale to separate Fig 2D and 2F?

Response: We have also detected the DNA and mRNA amount in brain, but no significant difference was observed between KO and WT mice (Response Fig. 5). According to the expression data shown in NCBI Gene, NR2F6 expression in brain is very low. Maybe it is the reason that we did not observe significant difference.

Fig. 2D show the expression of viral genes in liver tissues and Fig. 2F show the expression of anti-viral host genes.

8. Fig 3B: it is not clear if these critical data are from biological triplicates or no duplicates? RNA-Seq on single sample does not support any conclusions.

Response: As we claimed in the section of Method/Statistical analysis, two biological replicates were used here for RNA-Seq.

Response Fig. 5 DNA amount (A) and gene expression (B) of HSV-1 in the brain tissues of wild type and wild type and *Nr2f6*^+/-^ mice (n = 5, three tissues were measured for each mouse).

9. Fig 3C: It seems top hits are related to cell migration- how these processes are related to anti-viral responses and NR2F6 anti-viral function?

Response: It has been shown NR2F6 is also involved in tumorigenesis ^1,2^. Genes of cell migration are critical for tumorigenesis. We do not know whether the cell migration genes regulated by NR2F6 is involved in anti-viral response. Further experiments are required to determine it.

10. Fig 3E: to demonstrate cGAS/STING pathway is not involved, cGAS or STING knockdown should be performed in THP1 cells.

Response: We have performed experiments in a cGAS-KO L929 cell line as suggested. The results show that Nr2f6 knockdown also decreased the expression of viral gene US11 in cGAS-KO cells (Response Fig. 6).

11. Fig 4A: the knockdown efficiency of NR2F6 is quite low here.

Response: We have re-run the lysate and replaced the blot with a new one (Rev. Fig. 4B).

**Response Fig. 6 Nr2f6 knockdown repressed HSV-1 gene expression in cGAS KO L929 cells. (A)** cGAS knockout L929 cells were generated by CRISPR-Cas9. **(B&C)** cGAS knockout L929 cells were transfected with control or Nr2f6 siRNA for 48 h. The cells were infected with HSV-1 (MOI = 1) for 8 h before qRT-PCR analysis.

12. Fig 5B: low quality IF images didn’t add much to this study. Why not detecting endogenous NR2F6? If HSV-1 infection does not affect NR2F6 cellular localization, how does HSV-1 infection regulate NR2F6 function at enhancers?

Response: We did try the NR2F6 antibody in IF for the endogenous protein, which gave us also high background in knockdown cells. That’s why we only used it in the WB assays.

Based on our results, HSV-1 infection did not obviously affect NR2F6 localization, but inhibits its expression. Down-regulation of NR2F6 probably caused repression of its target enhancers, which led to the identification based on the repressed enhancers.

13. Fig. 5: a critical piece of information is missing here: does NR2F6 binds c-Jun or co-localizes with c-Jun by ChIP on super-enhancers defined in Fig 1? – NR2F6 ChIP-Seq would be necessary.

Response: We indeed had the same expectation before, and checked their potential interaction through immunoprecipitation. We successfully detected the interaction between the over-expressed proteins, as well as between over-expressed NR2F6 and endogenous c-Jun, but failed with the endogenous NR2F6 and c-Jun (Response Fig. 7). Since we are not sure about their interaction, we have not presented the data in the manuscript.

Since our antibody for NR2F6 does not work in ChIP-Seq analysis, we have performed Capture ChIP many times and tried to get sequencing data. The PCR results of 5 experiments were shown (Response Fig. 8). We selected DNA purified from 3 capture ChIP experiments for sequencing. Unfortunately, all the sequencing results gave us very high background. Then we took the overlapped peaks of the Capture ChIP and GEO NR2F6 ChIP-Seq, and analyzed their function (Response Fig. 9A&B). Interestingly, they are mostly enriched in the processes related with transport. When we overlapped the binding sites of NR2F6 and c-Jun, we found around 20% of c-Jun binding sites are overlapped with NR2F6 (Response Fig. 9C&D). We did identify the overlapped peaks on the potential enhancer region of MAP3K5 (Response Fig. 9E).

**Response Fig. 8 Capture ChIP in THP-1 cell stably expressing GFP-FB or NR2F6-FB.** Enrichment on MAP3K5 was detected by Q-PCR. Five experiments were shown. “With carrier DNA” means experometns were carried with addition of salmon sperm DNA.

**Response Fig. 7 Interaction between c-Jun and Nr2f6. (A)** HA-Nr2f6 and Flag-cJun were expressed in THP-1 and Streptavidin beads were used to pull down FB-tagged JUN. WB was performed as indicated. **(B)** THP-1 cells stably expressing HA-Nr2f6 were treated with/without HSV-1. Immunoprecipitation was performed with anti-HA and WB was done as indicated.

In conclusion, we found that NR2F6 and c-Jun possibly interact with each other and are co-localized on chromatin. We feel a better antibody might be important to confirm it.

**Response Fig. 9 Bioinformatic analysis of NR2F6 capture ChIP results. (A)** The target genes of NR2F6 Capture ChIP and GEO NR2F6 ChIP-Seq were overlapped, and their expression levels were shown by heat map. **(B)** The GO analysis of the above overlapped target genes. **(C)** Venn diagram to show the overlapped binding sites of NR2F6 and c-Jun. **(D)** The enrichment of NR2F6 on the overlapped binding sites shown in (C). **(E)** The IGV view of c-Jun and NR2F6 binding close to MAP3K5. Yellow color highlighted the overlapped peaks.

14. Fig 6B: why NR2F6 blot here becomes a doublet with a different pattern than other NR2F6 blots (eg. Fig 3 and Fig 4)?

Response: When we detect the protein level of endogenous NR2F6 with the antibody we have, we could often see two bands with long exposure, and only one band with short exposure. In Fig. 5C, we have shown that the major NR2F6 forms in cytosol and nuclei are different in molecular weight. It is possible that two different forms of NR2F6 exist in the cells, but currently we do not know whether they are different isoforms or with different modifications. Future studies are required to further elucidate the problem.

15. Fig 7F: why NR2F6 mRNA levels here in THP-1 cells upon HSV-1 infection remain the same (which is opposite to Fig. 1F (decreased) ?

Response: As we have shown in Fig. 6B, the mRNA level of NR2F6 decreases soon after HSV-1 treatment and recovers at 24 hr after infection. So, the time point to harvest cells for analysis determines whether the reduction of NR2F6 can be observed. In Fig. 7F, the cells were harvested 24 hr after infection and that’s why we did not observed NR2F6 decrease after HSV-1 treatment.

Reviewer #2: No major issues. The authors present a lot of data using complementary approaches that ultimately support the conclusions of the paper.

Response: We appreciate the reviewer for the positive comment.

Reviewer #3: (No Response)

Part III – Minor Issues: Editorial and Data Presentation Modifications

Please use this section for editorial suggestions as well as relatively minor modifications of existing data that would enhance clarity.

Reviewer #1: N/A

Reviewer #2: While I do not think any key experiments are missing, I think that the manuscript as it stands is unclear and difficult to get through due to presentation. Here are some comments and suggestions to help guide the authors to writing a clearer manuscript:

Response: Thanks for all the suggestions. We have tried our best to correct the manuscript.

• Why THP1 cells to start? This feels not particularly physiologically relevant considering the main latent reservoirs of HSV1 is neurons, and the general paradigm of disease involves initial infection at mucosal sites followed by neuroinvasion for establishment of latency. The manuscript would be greatly improved by either more clearly justifying the use of THP1s or performing more experiments in more relevant model cell types.

Response: Our main goal is to identify new transcription factors regulating innate immune response. Macrophage is one of main cell types responsible for innate immunity, and THP-1 is derived from monocyte and cGAS/STING pathway responds normal to DNA viruses. So, THP-1 cells are commonly used in the studies of innate immune response. In the current study, we have verified our results also in MEF, MLF, HFF, L929, BMDM, HEK293T and HACAT cells. The results all support our model. We have added the rational about cell line selection into the manuscript (line 116-117).

• In figure 1F, you show that late HSV1 gene expression is downregulated in the context of Nr2f6 KD, but it also looks like HSV1 infection leads to a decrease in Nr2f6 mRNA. MUCH later, in figure 6, you finally address this question in multiple cell types. I found myself thinking early on that it would be informative to know the dynamics of this protein vs RNA, especially since you’re saying it’s a transcription factor, thinking about the timing of expression to action. It might be useful to include the data from Fig6A-C early on, as early as figure 1 or 2.

Response: We understand the reviewer’s concerns about manuscript organization, and we had similar consideration when preparing the manuscript. The main goal for Fig. 1&2 is to demonstrate the function of NR2F6 in viral infection, and we do not want to distract the readers’ attention too much. During revision, we have added description about the mentioned point in the text of Fig.1F (line142). We hope the revision could meet the reviewer’s requirement.

• It would be extremely helpful if you reported more clearly the fold changes in RNAseq for the hits you’re following up on, especially Nrf26. Readers don’t want to have to dig through your whole dataset to see what this is. Especially because the directionality of change is not clearly noted for Nrf26, Figure 1E has a heat map with numbers but those numbers are not labeled so it is unclear what they mean.

Response: At the right part of Fig. 1E, a diagram about the relationship between the colors and expression levels has been shown, which is very common in the presentations of heat maps. For the details, the gene expression of all the genes are included in the Sup. Table 1&2.

• It would also be helpful to show where you found Nr2F6 in your ChIP seq early on – I found myself wondering where it was bound early on, and clearly you had seq data and then you validated it several figures later. I think it would be useful to show the tracks in Fig1 before following up on it rather than waiting till fig 5.

Response: The presented data in Fig. 1 is mainly about the approach of NR2F6 identification and validation, and the ChIP-Seq data used here is H3K27ac ChIP-Seq which is useful for the identification of enhancers and transcription factors. We are not sure whether it is helpful to move NR2F6 ChIP-Seq or ChIP data here. So we did not make changes about the comments.

• The focus in the text on “DNA viruses” when you are clearly only showing data for HSV1 is misleading and inaccurate. Especially when later you test an RNA virus – Sendai virus. This is quite cool! But it is not in line with the story you’re telling, and to suggest that what you’ve found for HSV1 is relevant for all DNA viruses, a viral lineage with immense diversity in disease, cell tropism, genome size and composition, and mechanisms of altering host cells to promote their infectious cycles, is not appropriate. Stay specific, or test another virus. It’s fine, interesting, and fun to speculate – would be great in the discussion! But you show no evidence for it here, yet, so framing your story this way in the introduction felt disingenuous.

Response: In Sup. Fig. S2E, we have also shown the effect of NR2F6 on MCMV, another DNA virus. Of course, we can not conclude that NR2F6 is involved in the responses to all DNA viruses. We have revised the description according to the suggestion (line 162-164). About SeV, although our data have shown NR2F6 is also involved in the response to it, we have not explored the underlying mechanisms yet. So in the current manuscript, we mainly focused on the regulation of NR2F6 to DNA viruses.

• You don’t mention the results for Fig 3 F ISG54 in the text at all – highlighting the difference between this and the other genes tested in this figure would help clarify your point

Response: We have cited it the results in the text (line 207-208). During revision, we have also edited the text to further clarify it (line 207).

• It would be extremely helpful to give a little more information about what Nr2f6 is earlier in the manuscript – the discussion has info that would be extremely useful to understand the rationale of experiments earlier on.

Response: We have added the description about NR2F6 in the section “Validation of transcription factors involved in anti-viral response” (line 147-150).

• The leftmost panel in Fig 1F is labeled “US11 level in MEF” but the y-axis is labeled “Relative virus DNA amount” – I’m pretty sure this is qPCR for viral genomes, but would be important to make sure these figures are labeled accurately. As a general note – I would recommend going through each figure to double check all of the labels for this type of mistake, and there are several figures that don’t have keys/labels in either the figure itself or the figure legend (Fig 1E, Fig 3C)

Response: Thanks for pointing out our mistakes. We have changed “Relative virus DNA amount” to “Rel. DNA amount of HSV-1 US11”, and checked the whole manuscript. We have added a label for Fig. 1E, and edited the legend for Fig. 3C.

• Fig 5F and 5H could use more informative labels than “ENCSR707QWA_1” etc – label them so readers can easily tell what they are looking at; same for Fig 6E, 7C.

Response: We have added more information into the labels, which should make it friendly for readers (Fig. 5F, 5H, 6E, 7C).

• What was the rationale for following up on Nrf26 and not Mef2d or Maff? Those data buried in the supplement are also compelling – especially that HSV1 seems to have the opposite effect on Maff (upregulated RNA levels) than it does on Nrf26. Perhaps this is will be a followup study? More clarity on the rationale of why Nrf26 was the focus would be helpful for following along in the story.

Response: We have added more description in the text about the rational to chose Nr2f6 for the following study (line 147-150).

• Sup Fig S1 “candidaters” typo – “candidates”

Response: We have corrected the mistake.

• I think if there’s a way to include it, I would have liked to see Sup Fig S4 in the main text – at least panels A-B. I understand space constraints, but this seems more important and interesting than relegating to supplement.

Response: We have moved Sup. Fig. S4A to Fig. 4A, and made the corresponding corrections.

• While the SeV work is interesting – what does it actually add to the story here? If you want to make the point that this could be a pan-viral effect of Nrf26, it would be more impactful to spend the whole start off the paper on HSV, and then at the end, have one figure with all of the Sev data that says look it is actually seemingly important for this RNA virus, too. That would leave the reader with excitement that you have found a potentially pan-viral function of this protein.

Response: I agree with the reviewer that it will have more impact if we could fully demonstrate the roles and mechanisms of NR2F6 in responses to RNA viruses. However, due to the limitation of funding and manpower, it will take us much longer to complete it. Currently, we only have our data for DNA viruses.

• Sup Fig S6 title is confusing. Directly target what?

Response: We have changed the title to “NR2F6 directly regulates the transcription of *UBC* and *TRIB1*”.

• The focus in the text on “enhancers” seems unnecessary – you did profiling of active chromatin marks, not just enhancers - or if you did, this was lost in the text.

Response: H3K27ac is usually distributed on promoters and active enhancers. For transcription factor prediction, it is important to just take the enhancer regions for analysis, which is relative far away from transcription start sites. We have edited the introduction to clarify it (line 102-104).

• The way you say NR2F6 is “a critical factor involved in anti-viral response” when your data show that NRF26 actually promotes HSV1 replication is confusing.

Response: We have changed the description to “a critical factor involved in viral infection and the interaction between viruses and host cells” (line 320-321).

• The last line “provides potential new targets for anti-viral drug design” feels not necessarily true. If you want to claim that, I think it would be important to at least speculate HOW in the discussion.

Response: We have removed the sentence (line 345).

Reviewer #3: I have the following comments that will improve clarity and readability:

1. Why were THP-1 cells chosen as a model system? Is this a good model for HSV-1 infection with MOI=1 infection? PMID: 30914680 implies that a high MOI should be used.

Response: THP-1 is one monocyte cell line, and can be induced to differentiate to macrophage. It also contains intact cGAS/STING pathway, which are deficient in man tumor cell lines. So, THP-1 is widely used in studying innate immune response to DNA viruses. The authors of the listed publication also used THP-1 for their study ^3^.

Different MOI is used for HSV-1 infection in different studies, mostly around 0.1-5 ^4-7^. We have used the number in the published studies ^8,9^. Higher titer might accelerate cell death and interfere the investigation of signaling pathways in some cell lines. The listed publication used MOI = 50 for study. I guess they maybe used a strain with less toxicity.

2. There is limited info on viruses used – i.e., what strain of HSV-1 was used?

Response: HSV-1 (KOS strain) was gifted by Dr. Hong-Bing Shu of Wuhan University, which was originally got from China Center for Type Culture Collection, Wuhan, China. We have added the information into the manuscript (line 383-384).

3. There are many instances of figures with multiple graphs/panels that are not referenced in the text and do not have figure legends. These are often easy to infer (i.e., a western of a knockdown), but there are several instances where it is not clear. I suggest giving each piece of data its own figure # (i.e. Fig. 1A/1B/1C). This issue (addition of appropriate figure legends and citation in the main text) should be rectified before publication. Some examples:

a. Figure 1E - what is the heat map showing? It’s not described in the figure legend

Response: We have added one title for the heatmap and edited the legend.

b. Figure 1F - the third graph is not described in the figure legend or referenced in the text.

Response: We have edited the legend to describe each panel of Fig. 1F.

c. Figure 1G – the right panel (US2 mRNA levels) isn’t referenced in the text and the western isn’t referenced in the figure legend or text.

Response: The legend was edited to reference all the panels in Fig. 1G. We have also gone through the legend to add necessary description.

3. A western of the knockdown of Nr2f6 should be shown in Figure 1. A western of knockdowns for Mef2d and Maff should be shown in the supplemental material.

Response: We have added the WB for Mef2d and Maff to Sup. Fig. S1. But the antibody we have in hand does not recognize mouse Nr2f6. We hope the reviewer could understand our difficulty. We have provided WB of NR2F6 for all the experiments done in human cell lines.

5. Some of the text is informal or needs clarification:

a. “DNA virus infections are detected in a large population of human beings and difficult to be cleaned due to its latent infection [1-3].” – “Cleaned” may be a typo for “cleared”; regardless, this is not true for all DNA viruses. The references cite only KSHV and HSV-1; ref. 1 does not seem appropriate for this statement.

Response: We have changed the sentence to “Infection of some DNA viruses, such as KSHV and HSV-1, are detected in a large population of human beings and difficult to be cleared due to its latent infection ” (line 63-65).

b. “Blank” is used in figures; I assume this refers to a mock infection so should be labeled “mock”

Response: We have changed it in the figures.

c. “It was quite difficult for us to get enough homozygous mice, and we guessed the homozygous may encounter problems in breeding or development.” Were any homozygous mice obtained? Is there another way to phrase this or quantify this problem? PMID: 18701084 – reported generation of homozygous knockout mice

Response: We did get homozygous KO mice. When we used the heterozygous mice for breeding, among the 51 pups, the number for +/+ : +/- : -/- = 14 : 31 : 6. Obviously, the number of homozygous does not meet the Mendel's law. The homozygous grow normally, but the females are difficult to get pregnant. When they get pregnant, usually each mother can only have 1-3 pups. Moreover, we find that even the heterozygous female mice slowly become difficult to get pregnant. We feel Nr2f6 KO causes some negative effects to animal breeding. That’s the reason we decide to use heterozygous for experiments. We know that other groups have reported the homozygous is normal and fertile, but it seems that we do encounter breeding problems for the homozygous. We guess it is maybe due to the difference of animal strains or knockout strategies.

d. “plus the fact that cGAS/STING pathway is defective in U2OS cells” needs a citation

Response: We have added the citation in the manuscript (line 198).

e. “All these were quite similar with NR2F6” is informal and should be rephrased

Response: We have changed the sentence to “The results were similar to those got from NR2F6 knockdown experiments” (line 226).

f. “Then through profiling of active enhancers, the change of active transcription factors is predictable in anti-viral response” is not clear and should be rephrased

Response: We have changed the sentence to “Then the transcription factors involved in anti-viral response, can be predicted through profiling of active enhancers, which may serve as a powerful tool to identify new regulators and pathways” (line 310-312).

6. NR2F6 looks like it is expressed at higher levels during HSV-1 infection in Fig. 5A. (MEF cells). Additionally, endogenous NR2F6 (the lower band) looks like it’s expressed at comparable levels during HSV-1 infection in Fig. 3E (right panel) and Fig. 4B (THP-1 cells). What do the authors attribute this difference to?

Response: We carefully checked the original data for Fig. 5A, and noticed that our labelling here caused some misunderstanding. The blot for Nr2f6 was still referring to the over-expressed HA-tagged Nr2f6, which was detected with NR2F6 antibody, not the endogenous Nr2f6. Actually, we failed to detect the endogenous mouse Nr2f6 protein with the current antibody. To avoid the misunderstanding, we removed the blot from Fig. 5A. In Fig. 3E and 4B, we thought the endogenous NR2F6 still decreased, although they were not as dramatic as those in other experiments. It is probably due to the variation of experiments which make the difference not as obvious as other results.

7. The difference in mobility between nuclear and cytoplasmic NR2F6 (only observable in Fig. 5C) is fairly subtle, and in the absence of additional data (identification of the PTM or validation in additional cell types), the claims surrounding this piece of data are quite speculative.

Response: We understand the reviewer’s concern. We have edited the text to ensure the accuracy of our description (line 243-246).

Small comments:

1. “LSD1, a histone H3K4 demethylase,” is a repetitive description of LSD1 from a few sentences earlier.

Response: We have deleted the words “a histone H3K4 demethylase” here (line 89).

2. Figure 1E - # of DEGs from 1B is 2959 (1065+1894); in the Venn diagram, there are 2956 (2933+23). Why the discrepancy?

Response: In the Venn diagram, a typo exists when we input the number. It should be 2936+23. We have changed the diagram (Fig. 1E).

3. Figure 2C legend: “treated with HSV-1, and infected with HSV-1” – what is “treated” referring to?

Response: We have deleted the words “treated with HSV-1” (line 800).

4. Fig 6A – what MOI/time points were used for infection?

Response: We used MOI = 1 here for all the cells. They were collected 8 hr after infection. We have also corrected the legend (line 865).

5. Fig. 7G – refer to ASK1 as MAP3K5 for consistency throughout the manuscript

Response: We have changed the gene name to MAP3K5 in Fig. 7G.

6. Are the same primers (i.e., for US2, US11) used for qPCR to quantify viral DNA replication as RT-qPCR?

Response: Yes, we used the same primers for qPCR and RT-PCR, but the protocols to prepare DNA or RNA were different.

**References**

1.	Kim, H. *et al.* Melanoma-intrinsic NR2F6 activity regulates antitumor immunity. *Science Advances* **9**(2023).

2.	Klepsch, V. *et al.* Nuclear receptor NR2F6 inhibition potentiates responses to PD-L1/PD-1 cancer immune checkpoint blockade. *Nature Communications* **9**(2018).

3.	Venuti, A. *et al.* HSV-1\EGFP stimulates miR-146a expression in a NF-kappaB-dependent manner in monocytic THP-1 cells. *Sci Rep* **9**, 5157 (2019).

4.	Li, C. *et al.* The RNA-binding protein LUC7L2 mediates MITA/STING intron retention to negatively regulate innate antiviral response. *Cell Discov* **7**, 46 (2021).

5.	Wei, J. *et al.* SNX8 modulates innate immune response to DNA virus by mediating trafficking and activation of MITA. *PLoS Pathog* **14**, e1007336 (2018).

6.	Zheng, Z. *et al.* Noncanonical autophagy is a new strategy to inhibit HSV-1 through STING1 activation. *Autophagy* **19**, 3096-3112 (2023).

7.	Ye, L. *et al.* USP49 negatively regulates cellular antiviral responses via deconjugating K63-linked ubiquitination of MITA. *PLoS Pathog* **15**, e1007680 (2019).

8.	Chen, L. *et al.* Inhibition of histone methyltransferase SETD8 represses DNA virus replication. *Cell Insight* **1**(2022).

9.	Gao, C. *et al.* The epigenetic landscapes of histone modifications on HSV-1 genome in human THP-1 cells. *Antiviral Res* **176**, 104730 (2020).
